# Editorial: The Need for Harmonised International Guidelines ahead of COVID-19 Human Infection Studies

**DOI:** 10.3389/phrs.2021.1603962

**Published:** 2021-01-29

**Authors:** Claas Kirchhelle, Samantha Vanderslott

**Affiliations:** ^1^ University College Dublin, Dublin, United Kingdom; ^2^ Oxford Martin School, University of Oxford, Oxford, United Kingdom; ^3^ Oxford Vaccine Group and Oxford Martin School, University of Oxford, Oxford, United Kingdom

**Keywords:** international guidelines, COVID-19, human infection studies, harmonisation, WHO, vaccine, human challenge studies

As evidenced by the October 2020 British go ahead for potential controlled human infection studies (CHIMs) (www.imperial.ac.uk/news/206893/uk-researchers-explore-human-challenge-studies/) and the publication of World Health Organization (WHO) criteria for COVID-19 CHIMs in May 2020 [1], the current pandemic may not only test the ethical limits of medical research but also open the door for much-needed globally-consistent guidance.

Also known as human challenge or infection studies, the idea behind CHIMs is to infect carefully monitored healthy volunteers with a pathogen to test novel treatments and vaccines. Rather than rolling out a new vaccine to thousands of people and relying on uncontrolled natural infections, CHIMs promise a much more rapid evaluation of efficacy in comparatively safe circumstances.

The history of CHIMs is closely tied to vaccine development. When English physician Edward Jenner developed a cowpox vaccine against the deadly smallpox virus in 1796, he decided to test its efficacy on his gardener's son, James Phipps [2]. Phipps received a cowpox inoculation and was later exposed to smallpox scabs. Phipps' lack of illness or characteristic smallpox scaring indicated that the new vaccine was effective. The advent of germ theory led to more systematic CHIMs. When British scientist Almroth Wright developed a vaccine against typhoid in 1896, he tested the vaccine by first inocculating himself and colleagues and then challenging at least one person with an injection of live typhoid bacteria [3]. Between 1946 and 1989, Britain's Common Cold Unit infected over 20,000 volunteers with common cold viruses [4]. Although the trials did not result in a proven vaccine, they improved knowledge of respiratory viruses and led to an anti-influenza compound [4]. Newer typhoid vaccines have also been tested with CHIMs, and CHIMs have been used to trial vaccines for other diseases like cholera and influenza.

There is clear evidence that CHIMs can speed-up research, but there are limitations to what information they can provide. Commentators have noted that using CHIM data from small groups of volunteers as a predictor of vaccine and therapy efficacy in the field is not straightforward: target populations will have varying microbiota, genetic profiles, and different co-morbidities like malnutrition, obesity, or burdens from other infectious or non-infectious diseases [5]. Small sample sizes will pose an inevitable problem for any CHIM involving COVID-19. Current plans for British CHIMs by Imperial College, the Department for Business, Energy and Industrial Strategy (BEIS), and clinical company (hVIVO) include a virus characterisation study and then studying how COVID-19 vaccines work. In the case of vaccines, it is, however, unclear how much additional scientific value these studies–which will be limited to healthy young volunteers aged between 18 and 30—will generate over large-scale ongoing phase III vaccine trials and post-rollout phase IV studies with tens of thousands of volunteers from all age groups across the world.

Ethically, COVID-19 infection studies are at the edge of what may be acceptable for CHIMs. Challenge studies require knowledge of the appropriate infectious dosage with which to test a vaccine and usually require a ‘rescue remedy’ if participants fall ill. The British CHIM proposal will gradually increase viral exposure to assess the minimum dose needed for an infection. However, there is still no known cure for SARS-CoV-2. The rescue-therapy Remdesivir has been found ineffective by the WHO’s ‘Solidarity Trial’ published in October (www.who.int/emergencies/diseases/novel-coronavirus-2019/global-research-on-novel-coronavirus-2019-ncov/solidarity-clinical-trial-for-covid-19-treatments). Historically, CHIMs were never employed for incurable diseases like rabies or Ebola [6]. In the case of the far less deadly Zika virus, US funders only recently decided that it was premature [6] to conduct CHIM-based vaccine studies. Some commentators argue that the current pandemic context justifies a revaluation of existing ethical limitations for COVID-19 CHIMs because of the comparatively low mortality risk for young adults. However, they remain vague on the cut-off point at which mortality risks become unacceptable–thus opening the door to a potentially dangerous weakening of existing ethical frameworks for CHIMs. Proponents have so far also failed to sufficiently address emerging research on ‘Long-COVID’, which indicates that the virus can cause long-term health damage in a significant group of survivors–including formerly young, healthy adults [7].

Deciding how to select participants will be of critical importance for the global acceptability of any future COVID-19 CHIM–regardless of where it takes place. The long and troubled history of medical experimentation on vulnerable populations calls for caution. In the 19th and 20th centuries, imperial powers trialled new vaccines and treatments on colonial subjects. Prisoners and racially and economically marginalized groups were also used as human guinea pigs. During the 1940s, US researchers infected four hundred prisoners with malaria to test potential treatments. In Nazi Germany, doctors conducted infection studies on concentration camp inmates [8]. Even after the 1947 Nuremberg Code established the principles of informed consent, voluntary participation, and freedom to leave a study, CHIMs in US prisons continued until 1976 [8]. Ensuring that COVID-19 CHIM participants fully understand both the mortality and morbidity risks resulting from a deliberate exposure to SARS-CoV-2 will be a major challenge.

Whether and how to reward participants is an equally difficult question. A recent survey at Oxford's Typhoidland exhibition (www.typhoidland.org) indicated that visitors favoured higher payments for CHIMS involving high-risk diseases. However, there are concerns about unduly influencing volunteers with high financial rewards and whether payments should be allowed to vary between identical CHIMs in high and low-income contexts.

Despite their chequered past and these important ethical questions, there is no harmonised international regulatory framework for CHIMs. In Britain, challenge agents are not considered drugs and studies do not require regulatory approval in addition to local ethics approvals if they do not include an investigational medicinal product. In the US, trials are subject to more stringent oversight by the Food and Drug Administration (FDA). In India, concerns about the ‘offshoring’ of clinical trials to poorer countries has led to clinical trials restrictions but legalising strictly controlled CHIMs is now [8].

The COVID-19 crisis offers an opportunity for the global community to rethink the patchwork approach to CHIMs. Properly designed CHIMs are an incredibly useful tool to aid medical research. The sheer scale of the current crisis justifies serious considerations of whether compromising the health of a small number of courageous volunteers is an acceptable trade-off for vaccines that will aid billions. Websites are asking for volunteers, and one group, ‘1Day Sooner’ (www.1daysooner.org) claims to already have found nearly 40,000 volunteers to take part in a CHIM against SARS-CoV-2.

However, in addition to the personal risks for volunteers, there is a danger that rushed, badly designed, or exploitative CHIMs can undermine wider trust in medical research. Past experiences like the 2019 CRISPR-baby scandal [9] show that weak standards in one place can bring disrepute on an entire community. Perceptions of divergent standards and rushed research can also undermine confidence in COVID-19 vaccines. While it is important to be wary of long-standing Western biases against research conducted by non-Western entities, allegations of a lack of transparency and insufficient or patchy trial data have marred the in-country launch of Russia’s ‘Sputnik V’ vaccine and the reputation of China’s vaccines (www.reuters.com/article/health-coronavirus-vaccines-attitudes/exclusive-international-covid-19-vaccine-poll-shows-higher-mistrust-of-russia-china-shots-idUSL8N2JQ17C).

Avoiding similar problems regarding CHIMs for COVID and other diseases is crucial. With the geographic locations of biomedical research becoming more diverse, the likelihood of pandemic events increasing, and the number of CHIMs rising since the 1990s, developing a common international framework for human challenge studies is highly desirable. Although local needs and constraints will vary, the WHO's eight criteria [1] for SARS-CoV-2 challenge studies, which include scientific justification and ethical assessment based on risks and potential benefits, are an important first step in what will hopefully become a wider push for harmonised global CHIM guidance. Trial participants, developers, and vaccine and therapy recipients in low-, medium-, and high-income contexts have much to gain from a more transparent global CHIM framework.

## Author Contributions

CK and SV conceived the idea for the paper, wrote and approved the final manuscript.

## Funding

Claas Kirchhelle receives funding from the Wellcome Trust and Samantha Vanderslott receives funding from the National Institute for Health Research (NIHR).

## Conflict of Interest

The authors declare that the research was conducted in the absence of any commercial or financial relationships that could be construed as a potential conflict of interest.

## Abbreviations

CHIM, Controlled human infection studies; COVID-19, Coronavirus Disease 2019; CRISPR, Clustered regularly interspaced short palindromic repeats; FDA, Food and Drug Administration; SARS-CoV-2, Severe acute respiratory syndrome coronavirus 2; US, United States; WHO, World Health Organization.

## References

[1] World Health Organization. “Key criteria for the ethical acceptability of COVID-19 human challenge studies” (2020). https://apps.who.int/iris/bitstream/handle/10665/331976/WHO-2019-nCoV-Ethics_criteria-2020.1-eng.pdf?ua=1. May 2020. (Accessed June 10, 2020).

[2] Riedel S Edward Jenner and the history of smallpox and vaccination. In: Baylor University Medical Center Proceedings, 18. Milton Park: Taylor & Francis (2005). p. 21–5. 10.1080/08998280.2005.11928028 PMC1200696 16200144

[3] Hardy A. "Straight back to barbarism": antityphoid inoculation and the Great War, 1914. Bull Hist Med. (2000). 74(2):265–90. 10.1353/bhm.2000.0073 10863829

[4] Lambkin-Williams R Noulin N Mann A Catchpole A Gilbert AS. The human viral challenge model: accelerating the evaluation of respiratory antivirals, vaccines and novel diagnostics. Respir Res. (2018). 19(1):123. 10.1186/s12931-018-0784-1 29929556 PMC6013893

[5] Shirley DA McArthur MA. The utility of human challenge studies in vaccine development: lessons learned from cholera. Vaccine (Auckl) (2011). 2011(1):3. 10.2147/VDT.S23634 24482781 PMC3904492

[6] Palacios R Shah SK. When could human challenge trials be deployed to combat emerging infectious diseases? Lessons from the case of a Zika virus human challenge trial. Trials (2019). 20(2):1–8. 10.1186/s13063-019-3843-0 31852506 PMC6921433

[7] McPartlin SO Morrison J Rohrig A Weijer C. Covid-19 vaccines: Should we allow human challenge studies to infect healthy volunteers with SARS-CoV-2?. bmj. (2020). p. 371, 10.1136/bmj.m4258; Available at: https://www.bmj.com/content/371/bmj.m4258 (Accessed: January 26, 2021). 33168564

[8] Jamrozik E Selgelid MJ. History of human challenge studies. In: Jamrozik, E., and Selgelid, Michael J., human challenge studies in endemic settings. Ethical and Regulatory Issues (Cham: Springer) (2021). 9–23. 10.1007/978-3-030-41480-1_2; Available at: https://link.springer.com/chapter/10.1007/978-3-030-41480-1_2 (Accessed January 26, 2021).

[9] Cyranoski D. The CRISPR-baby scandal: what’s next for human gene-editing As concerns surge after a bombshell revelation, here are four questions about this fast-moving field. Nature (2019). https://www.nature.com/articles/d41586-019-00673-1. (Accessed June 10, 2020). 10.1038/d41586-019-00673-1 30809070

